# Autonomic nervous system reactions to secondary exposure to disaster-related imagery

**DOI:** 10.3389/fpsyt.2026.1738606

**Published:** 2026-05-11

**Authors:** Chiaki T. Ono, Hironobu Kato, Yoshie Kikuchi, Zhiqian Yu, Yumiko Hamaie, Mizuki Hino, Kazuho Tomimoto, Hiroshi Komatsu, Saya Kikuchi, Yasuto Kunii, Tomohiro Uchida, Hiroaki Tomita

**Affiliations:** 1 Department of Psychiatry, Graduate School of Medicine, Tohoku University, Sendai, Japan; 2 Department of Regional Alliance for Promoting Liaison Psychiatry, Tohoku University, Sendai, Japan; 3 Department of Disaster Psychiatry, International Research Institute of Disaster Science, Tohoku University, Sendai, Japan; 4 Division of Psychology, Shokei Gakuin University, Natori, Japan; 5 Department of Preventive Medicine and Epidemiology, Tohoku University Tohoku Medical Megabank Organization, Sendai, Japan

**Keywords:** autonomic nervous system, disaster imagery, freeze response, Great East Japan Earthquake, heart rate variability, secondary exposure

## Abstract

**Aim:**

This study investigated how disaster-related imagery affects emotional and autonomic nervous system (ANS) responses, using heart rate (HR) and heart rate variability (HRV), in individuals with indirect exposure to the 2011 Great East Japan Earthquake (GEJE).

**Methods:**

Thirty-six healthy adults who had experienced strong ground shaking during the GEJE, but not the tsunami directly, viewed four types of videos: natural scenery (neutral), earthquake scenes, tsunami footage, and promotional videos repeatedly broadcast after the disaster. Subjective emotional responses (State-Trait Anxiety Inventory, Positive and Negative Affect Schedule), HR, and HRV indices were measured before, during, and after each video.

**Results:**

Compared to the neutral video, disaster-related videos significantly decreased HR and HRV during viewing, indicating an orienting or freeze-type ANS response. Earthquake footage, likely to evoke autobiographic fear, predominantly suppressed parasympathetic indices, while tsunami footage, associated with vicarious fear, predominantly suppressed sympathetic activity. Immediately after viewing, sympathetic activation increased significantly, consistent with a rebound active defense pattern. Notably, promotional videos did not induce subjective distress but still altered HR and HRV indices, suggesting unconscious physiological reactivity.

**Conclusion:**

Disaster-related imagery evokes distinct ANS responses depending on the emotional content and the viewer’s trauma history. Autobiographic and vicarious fear may differentially affect sympathetic and parasympathetic suppression, respectively. Furthermore, this cross-sectional evaluation demonstrates that even seemingly non-invasive media exposure years after a disaster can trigger autonomic changes. These findings underscore the urgent need for appropriate media broadcasting guidelines to protect public health following both seismic and climatic catastrophes.

## 1 Introduction

When large-scale disasters occur, extensive media coverage depicts the affected areas, often replacing regular television programming with disaster-related news broadcasts. As a result, a large proportion of the population is repeatedly exposed to disaster-related imagery. Numerous studies have examined the psychological effects of viewing such disaster-related media content (1–4), and several meta-analyses have further synthesized these findings (5, 6). While it is well established that direct trauma exposure adversely affects the psychosocial condition of the affected population (7–30), a growing body of evidence indicates that indirect exposure through the media also exerts significant negative psychological impacts (1–6). Rather than manifesting exclusively as clinical disorders, both forms of exposure frequently contribute to elevated depressive symptoms, acute stress reactions (ASR), and post-traumatic stress reactions (PTSR). These widespread symptoms, often occurring below the threshold of clinical diagnosis, pose a significant challenge to public mental health following disasters (7–28).

The 2011 Great East Japan Earthquake (GEJE) and subsequent tsunami devastated wide coastal areas of eastern Japan, resulting in more than 15,900 deaths and approximately 2,500 missing persons. Furthermore, the Fukushima nuclear power plant accident caused a profound and long-lasting impact on the lives of local residents. The mental health consequences of the GEJE have been reported extensively (7–30). Immediately after the disaster, images of the earthquake and tsunami were repeatedly broadcast on television. During the first month following the GEJE, regular television programs were canceled and replaced by news coverage containing footage of the disaster and affected areas, as well as a limited number of promotional videos produced by the Advertising Council Japan.

Even years after the disaster, GEJE-related footage continues to be aired during regular television programs and special broadcasts, particularly around the anniversary of March 11. In addition to television, people are frequently exposed to these images through the internet. Concerns have been raised that extensive exposure to traumatic imagery may secondarily traumatize individuals who have not directly experienced the disaster (31, 32). Conversely, secondary exposure has also been reported to facilitate post-traumatic growth (PTG), as observed among children living in disaster-affected areas. Positive attitudes toward memorial services and media coverage may promote PTG in children following natural disasters (11).

In reality, broadcasting disaster footage is unavoidable, and opportunities for exposure are numerous. It is therefore important to examine how such images affect physical and mental conditions, and to establish appropriate guidelines for broadcasting disaster-related footage and for advising viewers on how to engage with such content. These considerations are also critical for professionals who are routinely exposed to traumatic images. Although these images may evoke fear or anxiety, individuals sometimes fail to recognize their own emotional or physiological responses to the exposure. Such phenomena are recognized as emotional numbness or blunting, which is one of the major symptoms of acute or post-traumatic stress reactions (33–35). Therefore, to understand the nature of responses to disaster-related images, both subjective and objective indicators must be evaluated.

Exposure to aversive or disaster-related images can activate the body’s acute stress response pathways, even in the absence of a direct physical threat. When an individual perceives a threat through visual stimuli, the amygdala processes this information and signals the hypothalamus, which subsequently activates the autonomic nervous system (ANS) and the hypothalamic-pituitary-adrenal (PHA) axis. This activation leads to a cascade of physiological changes designed to prepare the body for survival.

The ANS is a critical component of the peripheral nervous system that regulates involuntary physiological processes, including heart rate, blood pressure, respiration, digestion, and metabolism. Regulation of these functions is essential for maintaining systemic homeostasis and enabling rapid adaptation to environmental changes. The ANS is divided into two major branches: the sympathetic nervous system, which mobilizes the body’s resources for “fight-or-flight” responses, and the parasympathetic nervous system, which promotes “rest-and-digest” functions and conserves energy. These sympathetic and parasympathetic branches interact dynamically to influence emotional, cognitive, and physiological processes (36).

The ANS is a critical component of the peripheral nervous system that regulates involuntary physiological processes, including heart rate, blood pressure, respiration, digestion, and metabolism. Regulation of these functions is essential for maintaining systemic homeostasis and enabling rapid adaptation to environmental changes. The ANS is divided into two major branches: the sympathetic nervous system and the parasympathetic nervous system. While traditionally conceptualized as opposing forces, with the sympathetic branch mobilizing resources for active defense (e.g., “fight-or-flight”) and the parasympathetic branch promoting restorative functions (e.g., “rest-and-digest”), modern psychophysiology recognizes their complex and dynamic interplay during threat responses. Notably, the parasympathetic nervous system is deeply involved in stress regulation; its rapid withdrawal initiates initial arousal, while its dominant activation in the face of certain threats can trigger a “freeze” response, characterized by profound physiological deceleration and immobilization. These sympathetic and parasympathetic branches interact dynamically to influence emotional, cognitive, and physiological processes (36).

The activities of the sympathetic and parasympathetic nervous systems are known to be closely related to stress conditions and intense emotions, such as fear and terror (37–39). Heart rate variability (HRV) has been widely used as an indicator of ANS function and is often employed to evaluate stress responses, emotional states, and mental health conditions (40–44). While much of the literature has focused on direct trauma, a growing body of research highlights the physiological impact of secondary exposure via broadcasting materials. Studies assessing subjective and physiological indices have demonstrated that viewing traumatic news broadcasts can elicit heightened cardiovascular reactivity and negative affect, mirroring mild stress responses.

A review by S. D. Kreibig summarized a wide range of findings on emotional responses and physiological indicators, demonstrating that heart rate (HR) responses differ not only between positive and negative emotions but also among specific types of negative emotions, such as anger, anxiety, disgust, fear, and sadness (38). Crucially, when individuals are exposed to aversive or threatening visual stimuli in a safe environment, their physiological reaction is strongly influenced by the “defense cascade” model (45, 46). According to this framework, the initial detection of a potential threat triggers an “orienting response” and a subsequent state of “attentive immobility,” which is commonly conceptualized as the initial “freeze” phase of the defense cascade. This stage is aimed at gathering information to appraise the danger, and is physiologically characterized by motor inhibition and parasympathetic dominance, leading to a transient deceleration in heart rate. However, if the threat is appraised as imminent or acts as a conditioned cue directly linked to past trauma, the cascade rapidly progresses to an active “fight-or-flight” defense, characterized by sympathetic arousal and heart rate acceleration.

Furthermore, several studies have reported distinct physiological responses to video-induced sadness and fear that align with this attentional framework. HR responses to video viewing appear to vary depending on the emotional content of the stimuli: decreases in HR have been observed during exposure to disgusting videos (e.g., blood/injury, medical, contamination-related content) (47), as well as following the viewing of videos that elicit negative emotions (48), reflecting this orienting and information-gathering process. In contrast, studies using fear-inducing videos have reported increases in HR, with the magnitude of the increase proportional to the intensity of subjectively experienced fear (49), reflecting a transition to a sympathetic active defense response. Collectively, these findings suggest that observed differences in HR responses may reflect qualitative differences in the emotional states elicited by video stimuli; however, this issue remains unresolved.

Importantly, the present study does not focus on individuals with severe direct trauma exposure, but rather on secondary exposure to disaster-related imagery through mass media, which is common even among those without direct life-threatening experiences. Therefore, this study aimed to investigate both subjective emotional responses and objective autonomic nervous system activity, indexed by HR and HRV, during and after exposure to earthquake, tsunami, and promotional footage from the GEJE.

Drawing on the defense cascade framework, we formulated hypotheses based on the participants’ specific disaster experiences. Because all participants directly experienced the severe tremors but not the tsunami, we expected these two types of footage to elicit distinct stages of the defense response (1). We hypothesized that earthquake footage, acting as a conditioned cue for autobiographical trauma, would rapidly bypass initial orienting and trigger a classic active defense response, characterized by sympathetic activation and significant subjective distress (2). In contrast, we hypothesized that tsunami footage, representing a massive, novel, and vicarious threat that participants did not experience directly, would prompt viewers to engage in intense information gathering to appraise the unfamiliar danger. Consequently, we expected this footage to elicit a profound orienting and “freeze” response, characterized by parasympathetic dominance and heart rate deceleration (3). Finally, we anticipated that promotional videos broadcasted repeatedly during the disaster period, while visually non-invasive, act as conditioned environmental cues. We hypothesized that these videos would induce subtle, unconscious physiological reactivity even in the absence of explicit subjective distress.

## 2 Materials and methods

### 2.1 Participants

Thirty-six healthy Japanese participants (11 males, 25 females), aged 20–34 years (mean age: 22.9 years), were recruited from Tohoku University and Shokei University in Miyagi Prefecture, Japan. Data collection was conducted between November 2014 and July 2015. At the time of the 2011 Great East Japan Earthquake (GEJE), these participants resided in eastern Japan and experienced strong ground shaking; however, they did not directly experience the tsunami or life-threatening damages. Thus, the present sample represents a population with indirect or low-level trauma exposure rather than direct disaster survivors. Participants were screened for post-traumatic stress symptoms using the Impact of Event Scale–Revised (IES-R), with a cutoff of >33 set as an exclusion criterion (50). None of the recruited participants met this criterion. The mean IES-R total score among participants was 8.75 (standard deviation [SD] =7.56).

The study protocol for data collection was originally approved by the Ethics Committee of Tohoku University Graduate School of Medicine prior to the experiment (Initial Approval No. 2013-1-081). Subsequent data analyses and minor administrative amendments, such as updates to the research team members, were conducted under the most recent continuous approval (Approval No. 2022-1-1138). The study complied with relevant Japanese laws, academic guidelines, and the Declaration of Helsinki. Written informed consent was obtained from all participants prior to their inclusion in the study.

### 2.2 Materials

#### 2.2.1 Video stimuli

Four types of video stimuli were used in this study: one neutral video and three disaster-related videos. Each video was compiled into a 5-minute segment. The disaster-related footage was adapted with permission from archive materials by Ad Council Japan, Higashi Nippon Broadcasting Co., Ltd., and Iwate Asahi TV Co., Ltd., licensed under Copyright © 2010 Ad Council Japan, Copyright © 2011 Higashi Nippon Broadcasting Co., Ltd., and Copyright © 2011 Iwate Asahi TV Co., Ltd., respectively. The videos included:

Neutral video (Baseline): Natural scenery, such as trees and floating clouds, serving as the baseline condition.

Earthquake video: Footage depicting earthquake-affected landscapes and individuals, including scenes captured at the exact moment of the earthquake accompanied by the Earthquake Early Warning alarm sound. It featured situations inside commercial facilities, evacuation from indoors to outdoors, and vehicles shaking violently outdoors.

Tsunami video: Footage showing large-scale destruction and survivors, including tsunami waves inundating port towns following tsunami warning sirens, people evacuating from approaching waves, and evacuees watching boats at sea after reaching higher ground. The footage did not include explicit scenes of individuals being swept away or deceased victims.

Promotional video: Public service announcements produced by AC Japan that were repeatedly broadcasted during the aftermath of the GEJE, a period when regular television programming and commercial advertisements were entirely suspended.

The video stimuli were presented on a 15.6-inch laptop display (Lenovo E540). To ensure consistency across conditions, all video files were rendered at a resolution of 640 x 480 pixels with a frame rate of 30 frames per second (fps). Participants viewed the videos seated at an approximate distance of 60 cm from the screen in a quiet laboratory room. Audio was delivered through the laptop’s built-in speakers at a comfortable listening level for all participants.

#### 2.2.2 Self-report questionnaires

Self-report questionnaires were used to screen participants and assess subjective emotional responses to the videos. Screening to assess baseline PTSD symptoms was performed using the Japanese version of the IES-R. The questionnaire consists of 22 items divided into three subscales: intrusion, avoidance, and hyperarousal. Items are rated on a Likert scale from 0 (not at all) to 4 (extremely) (50).

The emotional impact of video viewing was assessed using the followings:

Emotional Impact (PANAS): The Japanese version of the Positive and Negative Affect Schedule (PANAS) was used to evaluate emotional responses to each specific video. It consists of 20 items (10 for positive affect, 10 for negative affect) rated on a 6-point Likert scale. The instructions were modified to ask participants to rate their mood immediately after viewing each video (51).

Anxiety Levels (STAI-S): The state version of the State-Trait Anxiety Inventory (STAI-S) was used to assess acute anxiety. It consists of 20 items rated on a 4-point Likert scale (52).

### 2.3 Study design and procedures

Participants underwent a within-subject experimental protocol designed to assess psychological and autonomic responses to varying disaster-related visual stimuli.

After providing informed consent, participants were fitted with a heart rate monitor and remained seated comfortably throughout the experiment. First, they viewed the 5-minute natural video to establish a physiological a baseline. Following this baseline viewing, participants completed the demographic questionnaire, the IES-R, and the baseline STAI, and PANAS.

**Figure 1:**
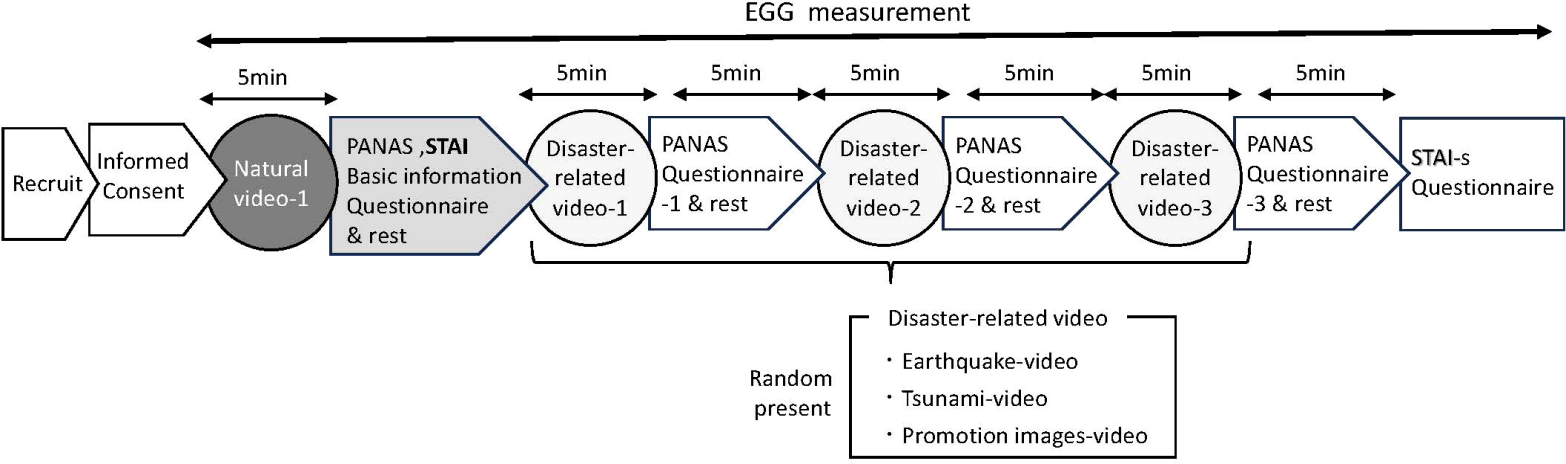
Diagram of the experimental setup and measurement protocol. Participants were recruited and, after providing informed consent, wore an electrocardiograph continuously until the end of the experiment. First, participants viewed a 5-min natural video and completed a questionnaire. They then viewed three types of disaster-related videos (earthquake, tsunami, and promotion), each lasting 5 min, presented in a randomized order. After each video, a 5-min rest period was provided, during which participants completed questionnaires. The PANAS was administered four times: after the natural video and after each disaster-related video. The STAI was administered twice: after the natural video and after completion of all video sessions.

Subsequently, participants viewed three disaster-related videos (earthquake, tsunami, and promotional footage). Each video lasted 5 minutes, and the presentation order was randomized across participants to pretend order effects. Immediately following each video, a 5-minute rest period was provided, during which participants completed the PANAS. After all three disaster-related videos and their respective rest periods were completed, participants filled out the STAI-S for second time to assess post-exposure anxiety (**Figure 1**).

### 2.4 Heart rate variability measurement

HR and HRV were assessed using Lead II electrocardiogram (ECG) signals recorded in a seated position (LRR-03; GMS, Tokyo, Japan). Frequency-domain HRV parameters were derived from the ECG signals and analyzed using the maximum entropy method (MEM) via Reflex Meijin software (CROSSWELL, Yokohama, Japan). Given the short-duration (5 minutes) and emotion-eliciting nature of the video task, autonomic regulation was primarily evaluated using frequency-domain indices, which are highly sensitive to rapid autonomic shifts.

HR: Heart rate (beats per minute).

HF (0.15–0.40 Hz): High-frequency power, reflecting parasympathetic nervous system activity.

LF (0.04–0.15 Hz): Low-frequency power, reflecting a combination of sympathetic and parasympathetic activity.

CCV-HF: Coefficient of component variance for HF, providing a normalized index of parasympathetic activity.

CCV-LF: Coefficient of component variance for LF.

LF/HF ratio: An indicator of sympathovagal balance, primarily reflecting relative sympathetic nervous system activation.

CVRR: Coefficient of variation of the R-R interval, indicating overall autonomic nervous system variability.

### 2.5 Statistical analysis

All statistical analyses were performed using SPSS Statistics software, version 29 (IBM Japan, Tokyo, Japan). Because the Shapiro-Wilk test revealed that several physiological variables were not normally distributed, non-parametric tests were employed for all analyses. To examine the overall differences in subjective and autonomic responses across the experimental conditions, a Friedman test was conducted. The within-subject factor was the video condition, comprising four levels: Neutral (Baseline), Earthquake, Tsunami, and Promotional videos. The dependent variables included the PANAS scores, HR, and all HRV indices (HF, LF, CCV-HF, CCV-LF, CVRR, and LF/HF ratio).When the Friedman test indicated a statistically significant main effect, *post-hoc* pairwise comparisons were performed using the Wilcoxon signed-rank test to compare the baseline against each disaster video condition, as well as to compare responses among the distinct disaster video conditions. To control for Type I errors in multiple comparisons, Bonferroni corrections were applied to the *post-hoc* p-values.

Additionally, the Wilcoxon signed-rank test (two-tailed) was used to compare HR and HRV indices measured during the video viewing versus after the viewing for each condition, as well as to compare STAI-S scores before and after the entire disaster-related video exposure protocol.

All statistical tests were two-tailed, and the significance level was set at *p* < 0.05.

## 3 Results

### 3.1 Demographic profile of participants

The demographic profile of the participants is shown in **Table 1**. All participants had lived in eastern Japan at the time of the GEJE. Although two participants had experienced severe damage to their homes, none had directly witnessed the tsunami, experienced life-threatening situations, lost family members due to the GEJE, or had a history of psychiatric illness.

**Table 1:** Demographic characterization of participants.

Variable Category	Data (N=36)
Sex	
Male/ Female	11 / 25
Age	
Mean(range)	22.9 (20-34)
location at the Time of the GEJE	
the Three Disaster-affected Prefectures (Iwate, Miyagi, and Fukushima)	21
Other Prefectures in East Japan	15
House destruction	
Completely destroyed	2
Extensively damaged	1
Moderately damaged	0
Slightly damaged	8
Minimal damage	18
No damage	7
Perceived Danger to Life	
Felt Great Danger	6
Felt Some Danger	16
Felt Little Danger	10
Felt No Danger at All	4

### 3.2 Changes in state anxiety and affective response

**Figure 2:**
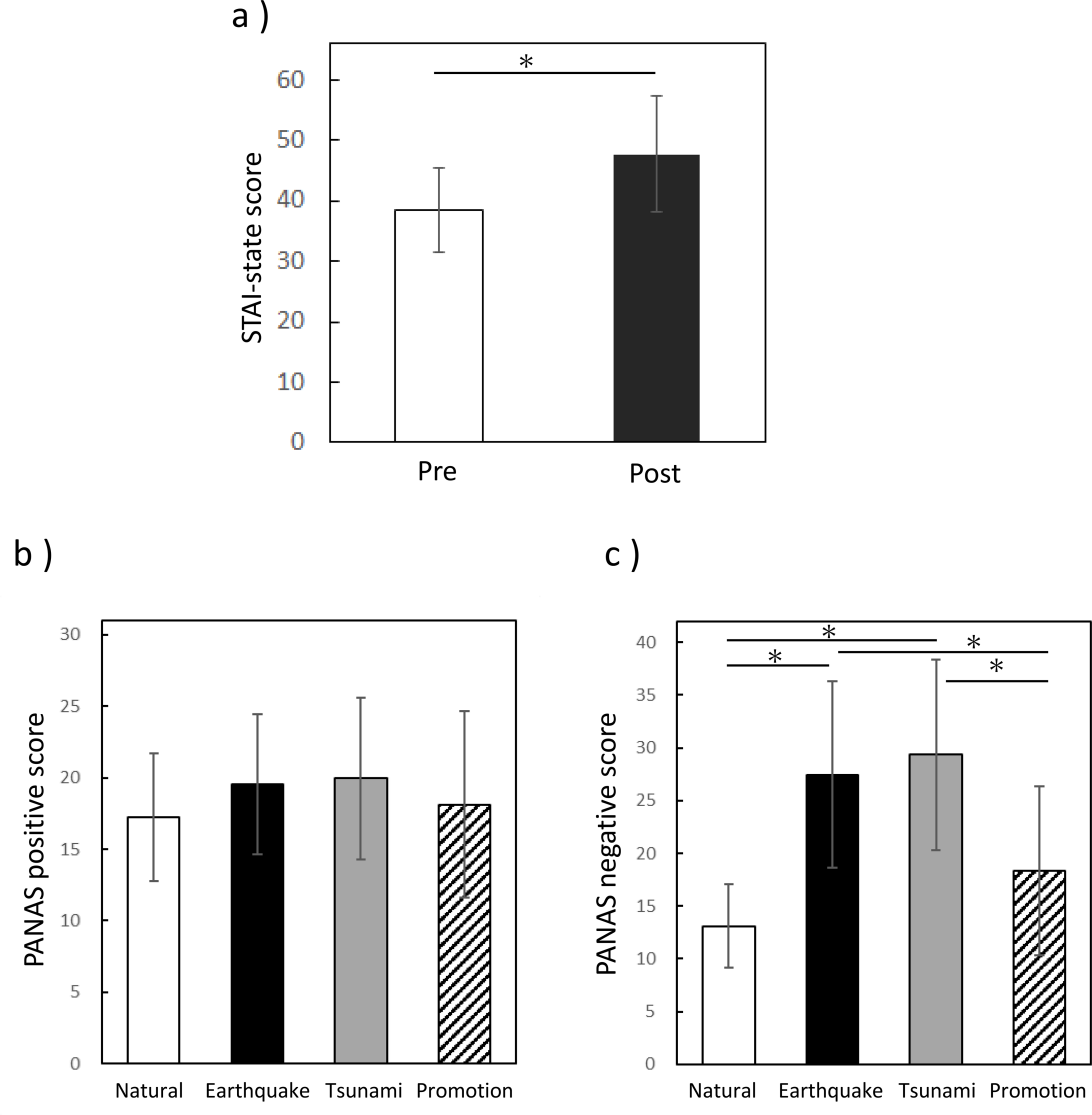
Psychological effects of viewing GEJE-related disaster videos. **(a)** State anxiety scores before and after viewing each video. **(b, c)** Positive and negative emotional responses to each video type: **(b)** total scores for positive emotions, **(c)** total scores for negative emotions. The bar graph shows the mean ± standard deviation (SD). Color coding: white, neutral video(n=35); black, earthquake video(n=36); gray, tsunami video(n=36); diagonal lines, promotional video (positive, n=36; negative, n=35). Statistical analyses were performed using the Wilcoxon signed-rank test and the Friedman test. Asterisk (*) indicate significant differences (*p* < 0.05).

To assess psychological reactions to disaster-related imagery, STAI-S scores were measured before and after the entire video session (**Figure 2a**). State anxiety scores were significantly higher after viewing all three disaster-related videos than after viewing the neutral video, as determined by the Wilcoxon signed-rank test (Z = -4.661, two-tailed, *p* < 0.0001). PANAS scores after each video condition were analyzed using the Friedman test. Significant differences were observed among video conditions for positive affect (X^2^ = 7.862, p < 0.05). However, *post hoc* pairwise comparisons with Bonferroni correction did not reveal significant differences between any of the videos. In contrast, negative affect differed significantly across conditions (X^2^ = 69.644, *p* < 0.0001). *Post-hoc* pairwise comparisons revealed that negative affect scores were significantly higher after viewing the earthquake and tsunami videos compared with the natural video (both adjusted *p* < 0.0001), whereas no significant difference was observed between the promotion video and the natural video. Furthermore, negative affect scores following the earthquake and tsunami videos were significantly higher than those following the promotion video (both adjusted *p* < 0.0001). (**Figures 2b, c**).

### 3.3 Physiological reactions during video viewing

#### 3.3.1 HR

**Figure 3:**
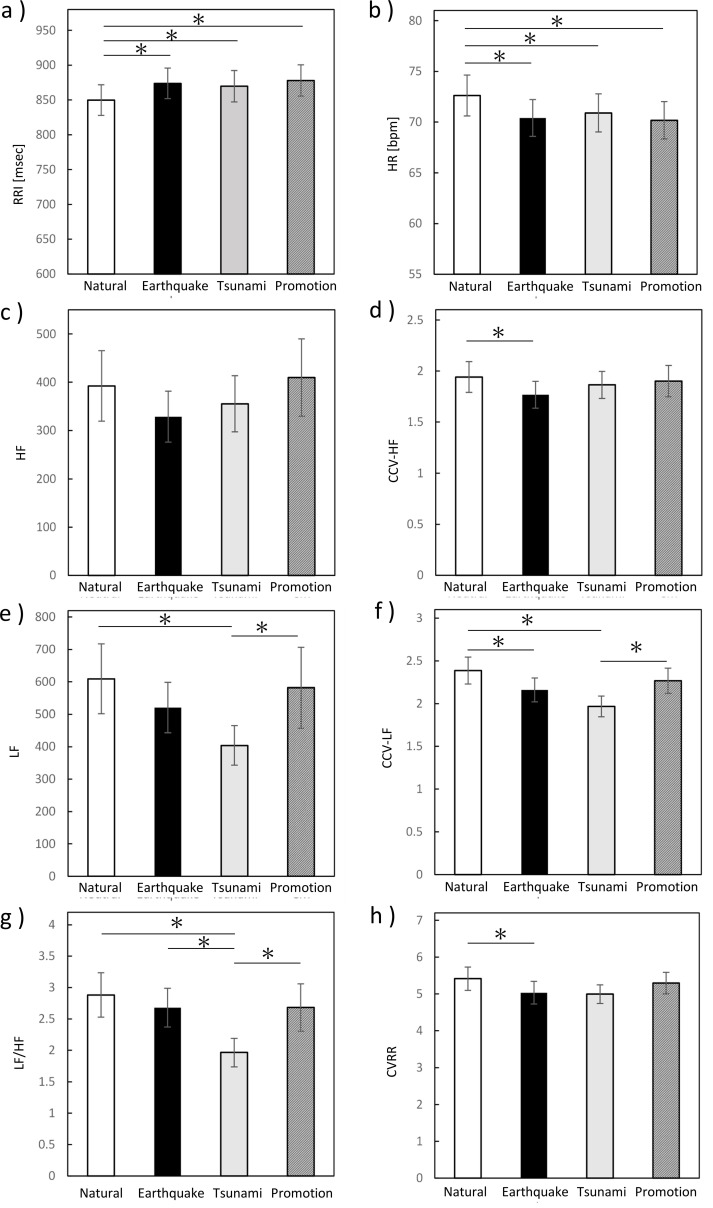
Autonomic nervous system responses during viewing of GEJE-related videos. **(a)** R–R interval (RRI), **(b)** heart rate (HR), **(c)** high-frequency power (HF), **(d)** coefficient of component variance for HF (CCV-HF), **(e)** low-frequency power (LF), **(f)** CCV-LF, **(g)** LF/HF ratio, and **(h)** coefficient of variation in the R–R interval (CVRR). The bar graph shows the mean ± standard deviation (SD). Color coding: white, neutral video; black, earthquake video; gray, tsunami video; diagonal lines, promotional video. Statistical analyses were performed using the Wilcoxon signed-rank test and the Friedman test. Asterisk (*) indicate significant differences (*p* < 0.05).

HR during the earthquake, tsunami, and promotion videos was significantly lower than during the neutral video, as determined by the Friedman test (X^2^ = 19.100, *p* < 0.0001) followed by *post-hoc* pairwise comparisons using Bonferroni correction (adjusted *p* = 0.002, *p* = 0.001, and *p* = 0.008, respectively). No significant differences were observed among the three disaster-related video conditions (all adjusted *p* = 1.000) (**Figures 3a, b**).

#### 3.3.2 Parasympathetic indices (HF and CCV-HF)

Among parasympathetic indices, a significant decrease in CCV-HF was observed only during the earthquake video compared with the neutral video, as determined by the Friedman test with *post-hoc* pairwise comparisons using Bonferroni correction (X^2^ = 8.833, *p* = 0.032; *post-hoc* adjusted *p* = 0.021). No significant changes were observed during the tsunami or promotion videos. HF exhibited a similar tendency during the earthquake video; however, this change did not reach statistical significance (**Figures 3c, d**).

#### 3.3.3 Sympathetic indices (LF, CCV-LF, and LF/HF)

Among sympathetic indices, LF values during the tsunami video were significantly lower than those during the neutral (adjusted *p* = 0.003) and promotion (adjusted *p* = 0.021) videos. Likewise, CCV-LF values during the tsunami video were significantly lower than those during the neutral (adjusted *p* = 0.001) and promotion (adjusted *p* = 0.021) videos. CCV-LF values during the earthquake video were also lower than those during the neutral video (adjusted *p* = 0.006). All comparisons were performed using the Friedman test (X^2^ = 14.267, *p* = 0.003 and X^2^ = 20.400, *p* < 0.0001 for LF and CCV-LF) with *post-hoc* pairwise comparisons using Bonferroni correction (**Figures 3e, f**).

Furthermore, LF/HF ratios during the tsunami video were significantly lower than those during the earthquake (adjusted *p* = 0.004), promotion (adjusted *p* = 0.008), and neutral (adjusted *p* < 0.0001) videos, as indicated by *post-hoc* pairwise comparisons following a significant main effect in the Friedman test (X^2^ = 19.433, *p* < 0.0001) (**Figure 3g**).

#### 3.3.4 Overall autonomic variability (CVRR)

CVRR values during the earthquake video were significantly lower than those during the neutral video, as indicated by *post-hoc* pairwise comparisons with Bonferroni correction following the Friedman test (X^2^ = 11.600, *p* = 0.009, adjusted *p* = 0.037; **Figure 3h**).

### 3.4 Autonomic nervous system fluctuations from during to after viewing

#### 3.4.1 HR

**Figure 4:**
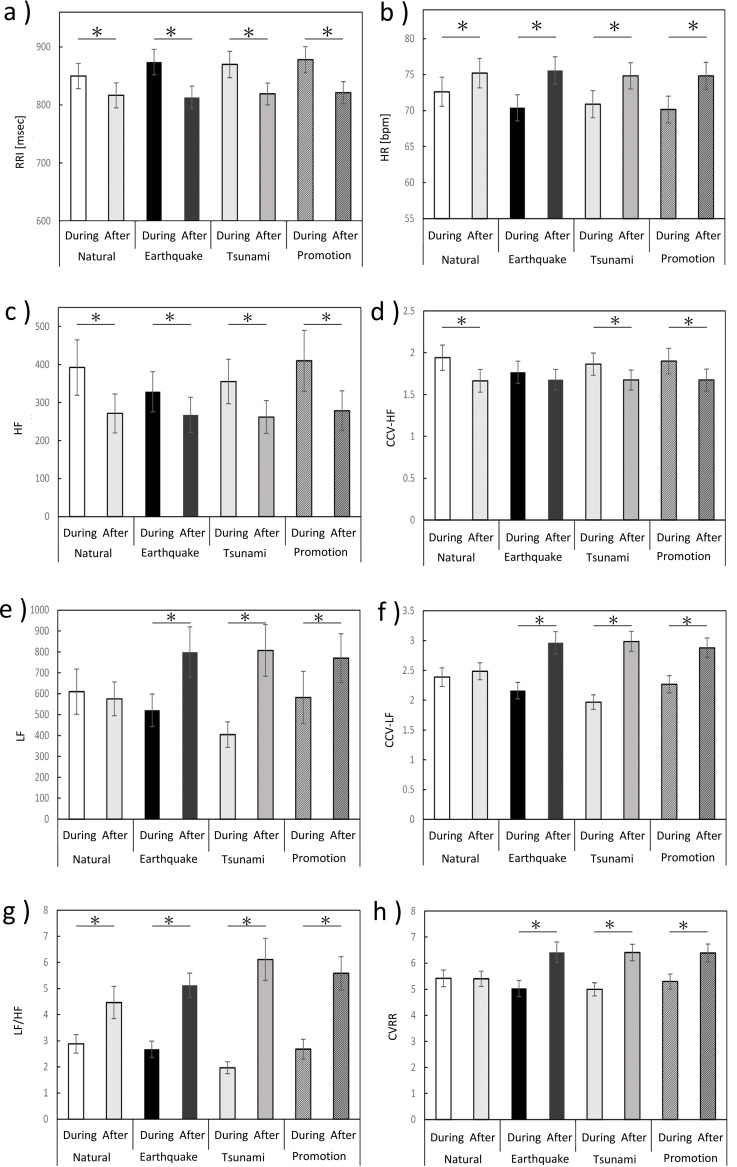
Autonomic nervous system responses during and after video viewing. **(a)** RRI, **(b)** HR, **(c)** HF, **(d)** CCV-HF, **(e)** LF, **(f)** CCV-LF, **(g)** LF/HF ratio, and **(h)** CVRR during and after each video session. The bar graph shows the mean ± standard deviation (SD). Color coding: white, during neutral video; light gray, after neutral video; black, during earthquake video; dotted black, after earthquake video; gray, during tsunami video; dark gray, after tsunami video; diagonal lines, during promotional video; black diagonal lines, after promotional video. Statistical analyses were performed using the Wilcoxon signed-rank test and the Friedman test. Asterisk (*) indicate significant differences (*p* < 0.05).

**Figure 5:**
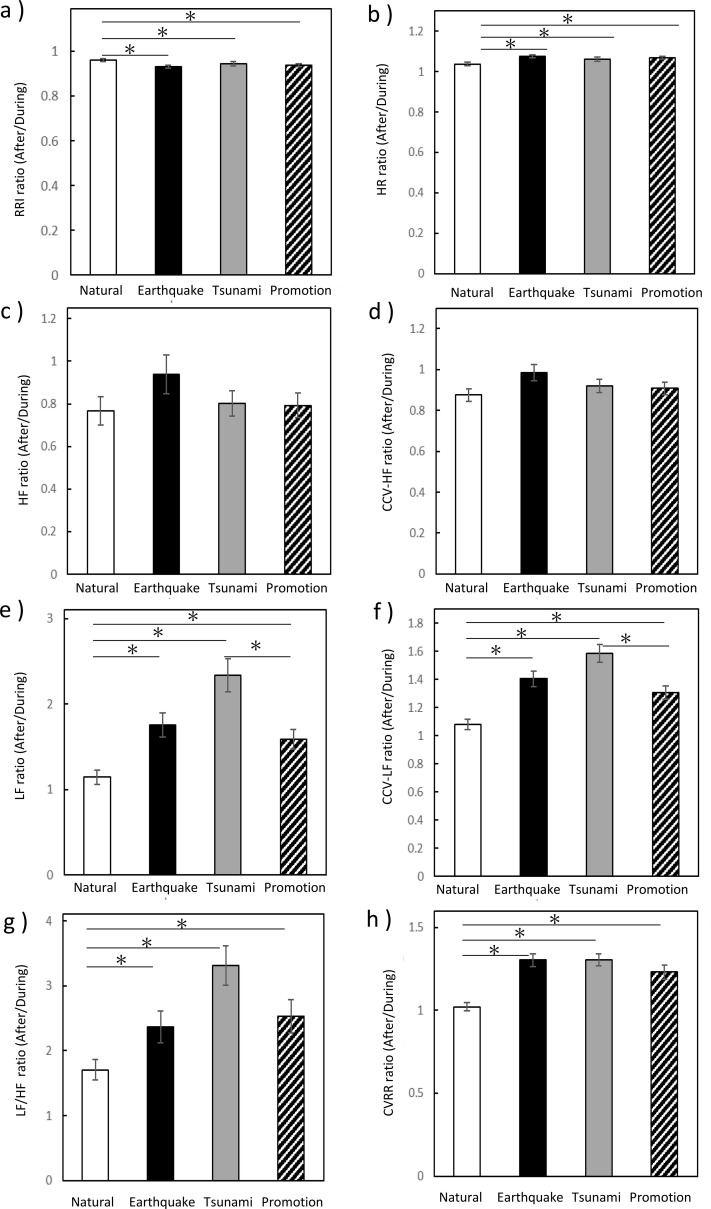
Ratios of autonomic nervous system activity after vs. during video viewing. **(a)** RRI, **(b)** HR, **(c)** HF, **(d)** CCV-HF, **(e)** LF, **(f)** CCV-LF, **(g)** LF/HF ratio, and **(h)** CVRR (each expressed as post-viewing/during-viewing ratio). The bar graph shows the mean ± standard deviation (SD). Color coding: white, neutral video; black, earthquake video; gray, tsunami video; diagonal lines, promotional video. Statistical analyses were performed using the Wilcoxon signed-rank test and the Friedman test. Asterisk (*) indicate significant differences (*p* < 0.05).

HR increased significantly after viewing all videos, including the earthquake, tsunami, promotion, and neutral videos, compared with during viewing, as determined by the Wilcoxon signed-rank test (neutral, Z=-3.865, earthquake, Z= -5.106, tsunami, Z=-4.242, promotion, Z=-5.090, all *p* < 0.0001; **Figures 4a, b**). However, the magnitude of this increase differed between the disaster-related videos and the neutral video. The post/pre increase ratios of HR were significantly higher for all disaster-related videos than for the neutral video, as assessed by the Friedman test (X^2^ = 23.833, p < 0.0001) followed by *post-hoc* pairwise comparisons with Bonferroni correction (earthquake, adjusted *p* < 0.0001; tsunami, adjusted *p* = 0.003; promotion, adjusted *p* = 0.001; **Figures 5a, b**).

#### 3.4.2 Parasympathetic indices (HF and CCV-HF)

HF and CCV-HF values decreased after video viewing compared with during viewing across all video conditions, except for CCV-HF following the earthquake video, as determined by the Wilcoxon signed-rank test (HF: earthquake, Z = -2.068, *p* = 0.009; neutral, Z =-4.415 tsunami and promotion Z =-4.006, all *p* < 0.0001; CCV-HF: earthquake, Z=-1.225, *p* = 0.220; neutral, Z =-4.148, tsunami, Z =-3.236 and promotion, Z =-3.362, all *p* < 0.0001; **Figures 4c, d**). However, no significant differences were observed in the post/pre decreased ratios of either HF or CCV-HF across video conditions, as assessed by the Friedman test (**Figures 5c, d**).

#### 3.4.3 Sympathetic indices (LF, CCV-LF, and LF/HF)

LF and CCV-LF values significantly increased after viewing all disaster-related videos (earthquake, tsunami, and promotion), but not after the neutral video, as determined by the Wilcoxon signed-rank test (LF: neutral, Z =-.597, *p* = 0.551; earthquake, Z =-4.320, tsunami, Z =-4.933, and promotion, Z =-4.210, all *p* < 0.0001; CCV-LF: neutral, Z =-.817, *p* = 0.414; earthquake, Z =-5.090, tsunami, Z =-5.153, and promotion, Z =-4.886, all *p* < 0.0001; **Figures 4e, f**). The post/pre increase ratios of both LF and CCV-LF were significantly higher for the disaster-related videos than for the neutral video. The Friedman test followed by *post-hoc* pairwise comparisons with Bonferroni correction revealed significant differences between the neutral and disaster-related conditions (LF (X^2^ = 35.900, p < 0.0001): neutral vs. earthquake, adjusted *p* = 0.003; neutral vs. tsunami, adjusted *p* < 0.0001; neutral vs. promotion, adjusted *p* = 0.049; CCV-LF(X^2^ = 48.033, p < 0.0001): neutral vs. earthquake, adjusted *p* < 0.0001; neutral vs. tsunami, adjusted *p* < 0.0001; neutral vs. promotion, adjusted *p* = 0.016). In addition, significant differences were observed between the tsunami and promotion videos for both LF (adjusted *p* = 0.006) and CCV-LF (adjusted *p* = 0.001; **Figures 5e, f**).

LF/HF values increased significantly after viewing all videos, including the earthquake, tsunami, promotion, and neutral videos, compared with during viewing, as determined by the Wilcoxon signed-rank test (all *p* < 0.0001; **Figure 4g**). However, the magnitude of this increase differed across video conditions. The post/pre increase ratios of LF/HF were significantly higher for all disaster-related videos than for the neutral video, as assessed by the Friedman test (X^2^ = 26.433, p < 0.0001) followed by *post-hoc* pairwise comparisons with Bonferroni correction (earthquake, adjusted *p* = 0.028; tsunami, adjusted *p* < 0.0001; promotion, adjusted *p* = 0.016; **Figure 5g**).

#### 3.4.4 Overall autonomic variability (CVRR)

CVRR values increased significantly after viewing all disaster-related videos (earthquake, tsunami, and promotion), but not after the neutral video, as determined by the Wilcoxon signed-rank test (neutral, *p* = 0.975; earthquake, tsunami, and promotion, all *p* < 0.0001; **Figure 4h**). The post/pre increase ratios of CVRR were significantly higher for all disaster-related videos than for the neutral video, as assessed by the Friedman test (X^2^ = 35.900, p < 0.0001) followed by *post-hoc* pairwise comparisons with Bonferroni correction (earthquake and tsunami, adjusted *p* < 0.0001; promotion, adjusted *p* = 0.002; **Figure 5h**).

## 4 Discussion

In this study, we found that disaster-related videos elicited characteristic autonomic responses that varied depending on the emotional content of the imagery. During video viewing, HR was lower and parasympathetic indices such as CCV-HF tended to decrease, while LF and CCV-LF were reduced particularly during the tsunami video. After video viewing, HR and sympathetic indices (LF, CCV-LF, LF/HF) increased markedly, with greater rebound responses following disaster-related videos compared to the neutral video as summarized in **Table 2**. In the following sections, we will discuss these physiological phenomena in more detail, addressing the distinct patterns observed during video viewing and the rebound responses after viewing.

**Table 2:** Summary of autonomic nervous system responses to disaster-related imagery.

Autonomic index		Neutral video	Earthquake video				Tsunami video				Promotion video			
		Δ (After – During)	vs. Neutral		Δ (After – During)	vs. Neutral	vs. Neutral		Δ (After – During)	vs. Neutral	vs. neutral		Δ (After – During)	vs. Neutral
			During	After		After/ during ratio	During	After		After/ during ratio	During	After		After/ during ratio
Cardiac Activity	RRI	↘	△	–	↘	▼	△	–	↘	▼	△	–	↘	▼
	HR	↗	▼	–	↗	△	▼	–	↗	△	▼	–	↗	△
Overall autonomic nervous	CVRR	–	▼	△	↗	△	–	△	↗	△	–	△	↗	△
Sympathetic and parasympathetic	LF	–	–	–	↗	△	▼	△	↗	△	–	△	↗	△
	CCV-LF	–	▼	△	↗	△	▼	△	↗	△	–	△	↗	△
Sympathetic	LF/HF	↗	–	–	↗	△	▼	△	↗	△	–	△	↗	△
Parasympathetic	HF	↘	–	–	↘	–	–	–	↘	–	–	–	↘	–
CCV-HF	↘	▼	–	–	–	–	–	↘	–	–	–	↘	–

Δ (Post - During) represents the physiological change from the viewing period to the post-viewing rest period.

Arrows indicate the direction of change within each condition: increased (↗), decreased (↘), or no significant change (−).

Triangles indicate the statistical comparison between each disaster-related video and the neutral video: significantly higher (△), significantly lower (▼), or no significant difference (-).

During: Comparison of absolute values during the video viewing period.

Ratio: Comparison of the post-viewing/during-viewing response ratios.

RRI, R–R interval; HR, heart rate; CVRR, coefficient of variation of the R–R interval; LF, low-frequency power; HF, high-frequency power; CCV-LF, coefficient of component variance for LF; CCV-HF, coefficient of component variance for HF.

### 4.1 Subjective emotional responses to disaster-related videos

After viewing all disaster-related videos, participants exhibited elevated levels of anxiety. Notably, negative emotions increased significantly during the earthquake and tsunami videos.

These findings align with previous reports showing elevated state anxiety following disaster video exposure, suggesting that not only direct disaster experience but also disaster-related visual media can significantly influence subjective emotional states. While previous studies using aversive stimuli or emotional film paradigms have often reported both decreases in positive affect and increases in negative affect following exposure, the pattern of subjective changes appears to vary across studies (53, 54). In the present study, positive affect did not significantly change, whereas negative affect increased significantly during the earthquake and tsunami videos. Such differences may partly reflect variations in stimulus content and the dominant emotional components elicited by the videos. Disaster-related footage may predominantly evoke high-arousal threat-related emotions (e.g., fear and anticipatory anxiety) rather than broad reductions in positive mood, thereby producing selective increases in negative affect without concomitant decreases in positive affect. Thus, the specific characteristics of the video stimuli may modulate the pattern of subjective emotional responses.

### 4.2 Heart rate deceleration and the orienting response

The earthquake and tsunami videos significantly decreased HR and indicators of sympathetic nervous system activity compared to the non-invasive neutral videos. According to the DSM-5, traumatic exposure is typically accompanied by strong emotions such as “fear,” “terror,” and “helplessness” (55). Additionally, traumatic events are often associated with a “fight-or-flight” response, which is closely linked to feelings of “surprise” and “excitement.” These emotions are commonly recognized as being associated with sympathetic nervous system activation. Therefore, the present finding that secondary exposure to traumatic imagery led to reduced HR and decreased sympathetic activity appears paradoxical at first glance.

However, this deceleration can be explained by attentional mechanisms. According to the intake–rejection theory proposed by Lacey et al. (56), HR decreases during the intake of external information. This cardiac deceleration has been interpreted as reflecting enhanced attentional engagement and sensory intake. In the context of threat perception, this aligns with the “orienting response” or “attentive immobility.” From this perspective, viewing natural and promotional videos may minimally induce an “intake” state characterized by sustained attention to external stimuli. However, the more pronounced decrease in HR observed during the viewing of earthquake- and tsunami-related videos likely reflects intense orienting, greater attentional capture, and deeper emotional processing elicited by these disaster-related stimuli. Previous studies examining the relationship between negative emotions and HR have shown that certain negative emotions, including disgust, fear of imminent threat, and acute sadness, can lower HR (38, 57). This suggests that HR may decrease in response to disaster imagery that evokes vicarious fear, threat, or intense sadness in a safe observational setting. Notably, it has been reported that sadness without crying decreases HR, while sadness accompanied by crying increases it, indicating that autonomic system responses may change depending on the depth and expression of emotion (38, 57).

### 4.3 Differential autonomic suppression: autobiographical vs. vicarious fear

It is noteworthy that during video viewing, the earthquake video predominantly suppressed parasympathetic nervous activity, whereas the tsunami video predominantly suppressed sympathetic nervous activity. All participants had been living in eastern Japan at the time of the GEJE, including those residing in areas closest to the epicenter (e.g., Miyagi Prefecture) as well as more distant regions such as Hokkaido and Yamanashi. Although all participants reported experiencing intense seismic shaking, rated between 4 and 6 on the Japanese seismic intensity scale, none had directly witnessed the tsunami (58).

Therefore, the earthquake footage likely triggered autobiographical recall of participants’ own fearful experiences, while the tsunami footage may have evoked fear by depicting threats to others rather than reactivating personal memories. The earthquake video primarily consisted of scenes showing people reacting to ground shaking, which was frightening but not portrayed as immediately life-threatening. In contrast, the tsunami footage depicted people fleeing from the approaching waves, which—despite omitting direct impact—likely conveyed a sense of life-threatening danger.

These distinctions suggest that re-experiencing one’s own fear is more strongly associated with parasympathetic suppression, while empathic fear through observation of others in danger may preferentially suppress sympathetic activity. Although both types of disaster footage suppressed overall ANS activity, the underlying mechanisms may differ depending on whether the viewer relates the content to personal trauma or to the observation of others in peril.

### 4.4 Interpretation within the defense cascade framework

Schauer et al. proposed a model of fear responses consisting of six linked defense cascade stages: freeze, flight, fight, fright, flag, and faint (59). They outlined corresponding patterns of HR changes associated with each stage. In stage 1 (freeze)—attentive immobility involving threat detection and evaluation—HR temporarily decreases. In stages 2 and 3 (flight and fight), HR increases as attempts to flee and fight response to perceived danger. These active defense stages are supported by sympathetic nervous system activation, which reorganizes blood flow via vasoconstriction of peripheral vessels and dilation of specific organ vasculature to supply the heart and skeletal muscles. Stage 4 (fright) marks a turning point in the autonomic response: peak sympathetic arousal is followed by parasympathetic activation. This peritraumatic state is characterized by co-activation of both systems and may resemble a panic-like physiological state. Stages 5 and 6 (flag and faint) represent a shutdown phase involving various forms of dissociation—such as altered sensory perception, motor function, and speech—which are associated with marked parasympathetic dominance and profound HR suppression.

Our findings may be cautiously interpreted within this defense cascade framework. While viewing a video in a safe laboratory setting does not constitute a direct life-threatening encounter and may not elicit a full biological defense cascade, HR during the viewing of GEJE earthquake and tsunami scenes was significantly lower than during neutral video viewing, indicating a “freeze-like” or orienting autonomic state. CCV-LF, an indicator reflecting combined sympathetic and parasympathetic activity, was also significantly reduced during earthquake and tsunami videos. These results suggest that both types of disaster-related footage suppressed overall ANS activity, indicating an attentive immobility or “freeze-like” autonomic state during exposure. To our knowledge, this is the first study to demonstrate that viewing disaster footage alone can induce a transient freezing response in the ANS of individuals indirectly exposed to traumatic events via imagery.

### 4.5 The post-viewing autonomic rebound

Immediately after participants stopped viewing the disaster-related videos, the ANS exhibited signs resembling a release from the freeze state and a shift toward the active defense stages (flight and fight). HR measured after viewing the disaster-related videos was significantly higher than those measured during viewing. The ratio of post- to during-viewing HR showed a significantly greater change following the earthquake and tsunami videos compared to the neutral condition.

Few studies have examined alterations in HRV between the periods during and after emotional video exposure. An exception is the study by Campbell-Sills et al., 2006 (60), who investigated this dynamic in individuals with mood and anxiety disorders. Participants were divided into two groups and shown emotionally evocative videos while being instructed either to suppress their emotions (suppression condition) or to accept and express them (acceptance condition). Interestingly, HR increased during viewing and decreased afterward in the suppression condition. Conversely, in the acceptance condition, HR decreased during viewing and increased after viewing—mirroring the pattern observed in the current study (60). Our study did not instruct participants to adopt a specific emotional regulation strategy, suggesting that participants may have been in a natural, acceptance-like state during video viewing, allowing for the full expression of the orienting deceleration followed by a physiological rebound.

Specifically, there were significant increases in overall ANS activity indicators (CVRR, LF, and CCV-LF) following the disaster-related videos compared to levels recorded during viewing. Furthermore, the ratios of post- to during-viewing CVRR and CCV-LF were significantly higher for the earthquake and tsunami videos than for the neutral video, reinforcing the notion of post-exposure activation.

The ratio of LF/HF, a marker of sympathetic nervous system activity, also increased significantly following the disaster-related videos relative to the neutral video. In contrast, the ratios of HF and CCV-HF, representing parasympathetic activity, did not show significant differences between disaster and neutral conditions. These findings suggest that the post-viewing increases in overall ANS activity were primarily driven by sympathetic activation (a rebound effect) following exposure to traumatic imagery.

### 4.6 Effect of repeatedly broadcast promotional videos on emotion and HRV

Viewing the promotional videos that were repeatedly broadcast in the aftermath of the GEJE did not elicit significantly different self-reported emotional responses on the PANAS compared to the neutral video. These promotional videos may no longer serve as strong triggers of negative emotions many years after the disaster. However, it is noteworthy that a discrepancy emerged between subjective emotional responses and physiological indices.

In the present study, HRV indices were significantly altered by these videos, even years after the GEJE. This suggests that the footage may have unconsciously activated disaster-related memories through classical conditioning, despite the content being non-invasive and not overtly distressing. Specifically, HR during the promotional video was significantly lower than during the neutral video. Additionally, LF/HF and CVRR values measured after viewing the promotional video were significantly higher than after the neutral video. These results indicate that even seemingly neutral, frequently rebroadcast disaster-related footage can activate the sympathetic nervous system, long after the original event. This emphasizes the importance of considering long-term physiological reactivity in individuals exposed to disaster-related media, even in the absence of consciously perceived emotional distress.

### 4.7 Limitations and future directions

There are several limitations to this study. First, the experiments were conducted between November 2014 and July 2015, approximately 3 years and 8 months to 4 years and 4 months after the GEJE. Individuals exposed to disaster-related imagery sooner or later after the event may exhibit different physiological or emotional responses.

Second, all participants were recruited from individuals who had lived in eastern Japan at the time of the GEJE. Because participants had limited direct exposure to the GEJE, the present findings should be interpreted as reflecting responses to secondary exposure to disaster-related imagery rather than the severe re-experiencing of personal trauma. Future studies should directly compare individuals with and without prior disaster experience (e.g., a completely non-exposed control group from a different geographical region) to clarify how personal exposure history modulates physiological and emotional responses to disaster-related media.

Third, the specific modality and quality of the video stimuli (e.g., screen size, resolution, and audio fidelity) may influence the degree of physiological engagement. Future research should investigate how immersive technologies, such as virtual reality or high-definition broadcasting, might amplify these autonomic responses.

### 4.8 Conclusion

In conclusion, while viewing earthquake and tsunami videos elicited significantly stronger subjective emotional reactions, they also suppressed global sympathetic and parasympathetic nervous system activity, reflecting an orienting or freeze-type response. The earthquake footage, likely to evoke autobiographical fear, more strongly suppressed parasympathetic activity, whereas the tsunami footage, conveying threats to others, more strongly suppressed sympathetic activity.

Immediately after viewing these images, significant increases in ANS activity were observed, primarily reflecting sympathetic activation consistent with a rebound flight-or-fight response. Although the repeatedly broadcast promotional videos in the aftermath of the GEJE did not elicit overt emotional distress, they significantly affected HR and HRV indices, even years after the event.

These findings suggest that both highly invasive and seemingly non-invasive disaster-related footage can provoke distinct and profound autonomic responses. As the global frequency of both seismic events and climatic catastrophes (e.g., severe floods and hurricanes) continues to rise, mass secondary exposure to such imagery is inevitable. This underscores the urgent need for careful consideration and the establishment of appropriate guidelines when designing post-disaster media communication strategies to protect public physiological and mental health.

## Data Availability

The raw data supporting the conclusions of this article will be made available by the authors, without undue reservation.
